# The Role of Cardiolipin and Mitochondrial Damage in Kidney Transplant

**DOI:** 10.1155/2019/3836186

**Published:** 2019-11-25

**Authors:** Alejandra Guillermina Miranda-Díaz, Ernesto Germán Cardona-Muñoz, Fermín Paul Pacheco-Moisés

**Affiliations:** ^1^Instituto de Terapéutica Experimental y Clínica del Centro Universitario de Ciencias de la Salud de la Universidad de Guadalajara, Guadalajara, Jalisco, Mexico; ^2^Departamento de Química del Centro Universitario de Ciencias Exactas e Ingenierías, Universidad de Guadalajara, Guadalajara, Jalisco, Mexico

## Abstract

Chronic kidney disease (CKD) is highly incident and prevalent in the world. The death of patients with CKD is primarily due to cardiovascular disease. Renal transplantation (RT) emerges as the best management alternative for patients with CKD. However, the incidence of acute renal graft dysfunction is 11.8% of the related living donor and 17.4% of the cadaveric donor. Anticardiolipin antibodies (ACAs) or antiphospholipid antibodies (APAs) are important risk factors for acute renal graft dysfunction. The determination of ACA or APA to candidates for RT could serve as prognostic markers of early graft failure and would indicate which patients could benefit from anticoagulant therapy. Cardiolipin is a fundamental molecule that plays an important role in the adequate conformation of the mitochondrial cristae and the correct assembly of the mitochondrial respiratory supercomplexes and other proteins essential for proper mitochondrial function. Cardiolipin undergoes a nonrandom oxidation process by having pronounced specificity unrelated to the polyunsaturation pattern of its acyl groups. Accumulation of hydroxyl derivatives and cardiolipin hydroperoxides has been observed in the affected tissues, and recent studies showed that oxidation of cardiolipin is carried out by a cardiolipin-specific peroxidase activity of cardiolipin-bound cytochrome c. Cardiolipin could be responsible for the proapoptotic production of death signals. Cardiolipin modulates the production of energy and participates in inflammation, mitophagy, and cellular apoptosis. The determination of cardiolipin or its antibodies is an attractive therapeutic, diagnostic target in RT and kidney diseases.

## 1. Introduction

Chronic kidney disease (CKD) is a serious public health problem in México and in the world [1]. The reports of the CKD registry in Jalisco (México) estimate the annual incidence rate of 411 per million inhabitants and the prevailing rate of 1556 per million inhabitants [2]. CKD has the capacity to progress to end-stage renal disease (ESRD) and to trigger serious complications that condition the death of patients. Cardiovascular disease (CVD) is the leading cause of death in patients with ESRD [3]. Hemodialysis (HD), peritoneal dialysis (PD), and renal transplantation (RT) constitute renal replacement therapy in ESDR [4, 5]. RT is the treatment of choice for patients with ESRD; however, the capacity of health systems worldwide is insufficient to meet the current needs [6]. It has been reported that the incidence of acute transplanted renal graft dysfunction ranges from 11.8% of related living donors to 17.4% of cadaveric donors [7, 8]. The existence of anticardiolipin antibodies (ACAs) may be a risk factor for renal thrombotic microangiopathy after RT. Asymptomatic ACA may be associated with acute graft dysfunction, even if thrombotic episodes are not observed [9]. Possibly, the early oxidation of cardiolipin can initiate the oxidation of low-density cholesterol (oxLDL), which could generate other inflammatory phospholipids [10]. OxLDL is abundantly found in atherosclerotic lesions and favors proinflammatory properties by activating T cells, endothelial cells, and monocytes/macrophages [11]. As reported, anticardiolipin antibodies (ACAs) are not associated with an increased risk of death among patients with HD or CVD [12]. Determining ACA and antiphospholipid antibody (APA) in candidates for RT can warn of possible early graft failure and indicate which patients can benefit from anticoagulant therapy [13]. In the present review, the cardiolipin in mitochondria and its effect on inflammation, oxidative stress, and mitophagy in CKD and RT are addressed. In addition, the role of ACA and APA in CKD and RT was reviewed.

### 1.1. The Nephron

The nephron is the functional unit of the kidney, whose main objective is to regulate the composition of body fluids through the processes of filtering, reabsorption, and secretion [14]. The active and passive solute reabsorption is carried out by the renal tubules in a process that consumes high levels of adenosine triphosphate (ATP) [15]. The production of ATP is mainly generated by aerobic metabolism [15]. Normal renal function involves numerous types of cells, including tubular epithelial cells, endothelial cells, and podocytes that work in coordination to form a delicate balance that involves many enzymatic processes and activities that require intensive energy use; among them was the transport of sodium that requires close coordination between supply and demand [16]. Renal tubular cells are rich in mitochondria and depend on the oxidative phosphorylation process for the generation of ATP for proper functioning. In case of mitochondrial injury or failure, renal cell functions are severely affected [17]. Even at rest, the metabolic rate of the kidney is high; a large number of functional mitochondria are required to provide enough energy to regulate fluid and electrolyte balance, maintain acid-base homeostasis, regulate blood pressure, eliminate waste from the bloodstream, and reabsorb the nutrients. Glomerular filtration is a passive process that depends on the maintenance of hydrostatic pressure in the glomeruli [18]. In contrast, the reabsorption of glucose, ions, and nutrients is driven by ionic gradients and occurs through ATP-dependent channels and transporters [19, 20]. The mitochondria are the main source of generation of reactive oxygen species (ROS) and the internal mitochondrial membrane respiratory complex. Complexes III and I are the main sites for the generation of the superoxide radical anion (O_2_^·-^) [21]. Cytochrome c (cyt c) catalyzes the oxidation of cardiolipin in the presence of ROS, which contributes to permeabilization of the mitochondrial membrane [22]. In addition, it has been reported that oxidation of ROS-induced cardiolipin affects the activity of respiratory chain complex I [23].

### 1.2. Cardiolipin

Cardiolipin (1,3-bis(sn-3′-phosphatidyl)-sn-glycerol) plays an important biological role by associating with subcellular particles of mitochondria [24]. Cardiolipin is a tetra-acyl anionic phospholipid highly conserved in bacteria and mitochondria, it is a dimeric phosphatidic acid with glycerol, and characteristically it contains a central prochiral carbon, two phosphates, and four acyl chains. Cardiolipin shows asymmetry in relation to the chains of acyl groups and their distribution in the internal mitochondrial membrane [25]. Cardiolipin phosphate groups were originally described as strong acids due to their pK values. Phosphates are ionized at a physiological pH, which makes cardiolipin an amphiphilic lipid with a negative net charge [26]. It was previously described that cardiolipin could have a pK1 close to 3 and a pK2 > 7.5. However, recently by calorimetric and pH titrations using cardiolipin with uniform saturation and fatty acid length chains, a pK1 = 2.15 and a pK2 = 3.15 were detected [27]. This is very important because the ionization state of cardiolipin influences (1) its interaction with other molecules in the mitochondrial inner membrane, (2) the membrane structure, and (3) the transport of molecules and ions [28]. Structurally, the four-acyl chains of the cardiolipin together with its negatively charged polar part give the cardiolipin a conical shape, the polar region being the top of the cone and the flexible and variable acyl chains the base of the cone. Cardiolipin spontaneously forms inverse laminar or hexagonal structures depending on the saturation of the acyl chains and the pH of the solution. Cardiolipin is crucial to maintain the curvature of the mitochondrial ridges and the potential of the inner membrane. The length, saturation, and oxidation of acyl groups provide differential influences on the shape, binding, function, and stability of cardiolipin [29]. Cardiolipin participates in the maintenance of the structural integrity of the mitochondrial membrane. The highest concentration of cardiolipin is concentrated in the inner monolayer of the inner mitochondrial membrane, and a significant portion binds to proteins. The enzymatic respiratory complexes of the mitochondrial respiratory chain, the adenine nucleotide transporter, the F_0_F_1_-ATP synthase, and decoupling protein 1 bind with high affinity to the cardiolipin for the correct assembly and stability of its quaternary structure and its correct functioning [30]. Each species of cardiolipin has subtle structural differences, and polyunsaturated acyl chains are candidates for lipid peroxidation. Like their unsaturated phospholipid equivalents, many cardiolipin oxidation products have the potential to transmit information to lipoperoxidation product receptors. Acyl fragments of the oxidized cardiolipin chains are capable of transmitting information upon dissociation [31]. The interactions of cardiolipin with proteins are essential to understand many of its functions. It has been documented that many proteins interact structurally and functionally with cardiolipin and their presence is essential for many of them [32]. Cardiolipins bound to respiratory complexes III and IV are crucial for electron transport. The cytochrome bc1 cardiolipin binding sites are located on the surface exposed to the membrane of respiratory chain complex III [33]. To maintain the integrity of the structure of complex III, the cardiolipin spontaneously diffuses at the interface of the cytochrome bc1 dimer that postulates the separate reduction of the quinone and the oxidation of the quinol site by completing a catalytic cycle, a site that receives a quinone molecule, two heme b electrons, and two protons from the negative membrane site, which also suggests that cardiolipins have an important role in the proton transport [34]. Cardiolipin molecules are strongly linked to cytochrome c oxidase (cyt c oxidase), a terminal complex of the respiratory chain essential for their function [35]. In fact, the sequential elimination of 3-4 cardiolipin molecules strongly bound to each cyt c oxidase monomer leads to the loss of electron transport activity and favors the dissociation of the VIa and VIb subunits of the enzyme complex [36]. Two cardiolipin binding sites in cyt c oxidase are involved in the pathways that bind cardiolipins to the entrance of proton D and H channels, highlighting the importance of cardiolipins in proton delivery to complex IV [37]. Other peripheral membrane proteins and soluble proteins interact with cardiolipin. For example, light chain 3 of the 1A/1B proteins associated with microtubules is involved in the mitochondrial degradation process (mitophagy) by recognizing the externalization of cardiolipin by damaged mitochondria [38]. In addition, cardiolipin participates in the maintenance of the nonspheroidal membrane structure [39]. Cardiolipin and its oxidation products should be recognized as cellular signals that regulate various metabolic pathways [40]. Polyunsaturated acyl chains of cardiolipin after oxidation produce countless lipid mediators of cell signaling [41]. The initially synthesized mitochondrial cardiolipin may include a variety of acyl chains. However, in various tissues, including the kidney, the nascent cardiolipin undergoes further transformations by a remodeling process to form cardiolipins with identical acyl chains. It is believed that excessive production of O_2_^·-^ radicals by the mitochondrial electron chain and by dehydrogenases of the Krebs cycle can produce random reactions of chemical oxidation in different biomolecules, including phospholipids that generate highly diversified molecular speciation of cardiolipins [42].

### 1.3. Mitochondria and Cardiolipin

Mitochondria are very varied complex and dynamic subcellular compartments; in a specific tissue, mitochondria can exist as small individual organelles or form a large tubular network attached to an interconnected membrane depending on environmental factors. The fundamental functions of mitochondria are cellular respiration, calcium homeostasis, phospholipid synthesis, fatty acid oxidation, apoptosis, ROS generation, and detoxification, among others [43]. Mitochondria contain four compartments: the outer membrane, the inner membrane, the intramembrane space, and the matrix. The outer membrane surrounds the organelle, is relatively permeable, and houses protein complexes that regulate the importation of proteins. The inner membrane is impermeable to ions and molecules, which is critical to maintain the electrochemical gradient that drives oxidative phosphorylation, is aligned adjacent to the outer membrane, and is characterized by the presence of numerous ridges that are divided into two distinct regions: the inner boundary membrane (IBM) and the ridges. The surface area of the inner membrane (IBM plus ridges) is >4 times larger than that of the outer membrane [44]. The expansion and contraction of ridges are physiologically relevant processes that occur in response to metabolic disturbances [45]. *In vitro* studies show that in giant unilamellar vesicles (GUV), cardiolipin induces the formation of narrow and dynamic tubular extensions or invaginations similar to mitochondrial ridges that can change shape according to the pH of the medium. Therefore, cardiolipin plays a crucial role in the formation of GUV-like invaginations; this effect may be relevant for the biogenesis of mitochondrial ridges [46]. For survival and interaction with the environment, cells require continuous production of ATP. The oxidative phosphorylation system produces 90-95% of ATP for tissues and organs that require high energy demand such as the brain, heart, and kidney. ATP is generated through the gradual oxidation of carbohydrates, amino acids, and fatty acids. For that reason, mitochondrial dysfunction is strongly associated with aging and many complex diseases [47]. Cardiolipin is crucial for the normal bioenergetic processes of mitochondria. It seems that peroxidation and cardiolipin depletion contribute to age-related mitochondrial dysfunction [48]. The structure of cardiolipin contributes to the formation of mitochondrial ridges, and it has been proposed that cardiolipin phosphate groups located in the outer monolayer of mitochondrial ridges trap protons [49]. Cardiolipin participates in the transformation of the proton gradient into an essential transmembrane electrochemical potential to maintain the synthesis of ATP by F_0_F_1_-ATP synthase [49]. Cardiolipin also acts as a scaffold for the correct assembly of respiratory complexes and supercomplexes, which facilitates the optimal transfer of electrons in the mitochondrial inner membrane [50]. Cardiolipin plays an important role in anchoring cyt c to the inner mitochondrial membrane and facilitating the transfer of electrons from complex III to complex IV [51]. Coenzyme Q within the internal mitochondrial membrane facilitates the transfer of electrons from complex I to complex III, while cyt c mediates the transfer of electrons through its heme that changes between the reduced ferrous state (Fe^2+^) and the oxidized ferric iron (Fe^3+^) [52]. Met 80 and His 17 are axial ligands of heme iron in cyt c, and they are also essential to stabilize the native conformation and electron transfer [53]. To support the transfer of electrons from complex III to complex IV, cyt c must be very close to these complexes. Therefore, the cardiolipin provides an anionic platform for electrostatic interaction with highly cationic cyt c (9+ net charge), which makes cyt c slightly attached to the electron transport chain. The electrostatic interaction of cyt c with cardiolipin is physiologically compatible with high levels of ATP synthesis [54]. When the concentration of ATP decreases in pathological conditions such as CKD, cyt c is closely associated with cardiolipin through hydrophobic interactions. The interaction of cardiolipin with cyt c leads to the disruption of the Fe-Met 80 bond, so the cyt c/cardiolipin complex acquires peroxidase/oxygenase activity [55]. The peroxidase activity of the cyt c/cardiolipin complex is favored in the presence of ROS and contributes to the oxidation of cardiolipin by facilitating the opening of the transition pore of mitochondrial permeability (mPTP) and the release of cyt c to the cytosol and triggering apoptosis. Therefore, cyt c has two contrasting functions, and its predominant activity is determined by oxidative stress and its interaction with cardiolipin [56]. In mitochondria, lipid peroxidation particularly affects cardiolipin that is expressed exclusively in the inner mitochondrial membrane and that plays a central role in preserving mitochondrial structure and function. Mitochondrial fission factor (Mff) deficiency reduces cytoplasm leakage in the cytoplasm relieving cardiolipin oxidation resulting from damage to electron transport chain complexes and overproduction of mitochondrial reactive oxygen species [57]. Under physiological conditions, cardiolipin strongly traps cyt c within the inner mitochondrial membrane. However, selective peroxidation of cardiolipin facilitates the release of cyt c from the outer surface of the inner mitochondrial membrane by opening the mPTP and its subsequent release to the cytoplasm by blocking oligomerization of the dependent anion of channel 1 voltage and the subsequent separation of hexokinase 2 from the mitochondria. It is not clear whether the fission associated with Mff triggers the opening of mPTP and the leakage of the cytoplasm by regulating the dissociation of hexokinase 2 and the oxidation of cardiolipin. If that happens, it is important to know which molecule links fission to changes in the release of hexokinase 2 and the peroxidation of cardiolipin. For example, during an acute traumatic brain injury, a significant number of mitochondria are released in the systemic circulation. These particles are highly procoagulant through the cardiolipin exposed to the surface [58]. A similar event could happen in the process of ischemia/reperfusion in RT. Oxidative stress is an event that arises from the reperfusion of an organ after a long period of ischemia and that leads to tissue damage. Reperfusion injury is a major problem in organ transplantation. In RT, the increase in reactive oxygen species (ROS) causes a delay in graft function by affecting the energy metabolism of renal cells. ROS-induced ischemia/reperfusion injury has a common substrate: the transition of mitochondrial permeability that causes permeability of the internal mitochondrial membrane after massive accumulation of Ca^2+^. The increase in nonspecific permeability occurs after the opening of a nonspecific pore with a diameter of approximately 2-3 nm that allows the release of the matrix content with a molecular weight of up to 1500 Da [59].

### 1.4. Cardiolipin and ATP Synthesis

Cardiolipin binds with high affinity to three sites in the mitochondrial carrier ADP/ATP. The conservation of amino acid residues at cardiolipin binding sites to other members of the mitochondrial transporter superfamily suggests that cardiolipin binding could be important for the function of all mitochondrial carriers [60]. Cardiolipin modulates mitochondrial functions, including the synthesis of ATP; however, the biophysical mechanisms by which cardiolipin generates nonlamellar structures and the degree to which these structures contribute to the synthesis of ATP are still unclear [61]. ATP synthase is a key enzyme for the conversion of mitochondrial energy, and the alteration of its activities is associated with several acute and chronic pathologies such as CKD [62]. Mitochondria provide energy to the Na^+^/K^+^-ATPase enzyme to generate ion gradients across the cell membrane. Because proximal renal tubules contain more mitochondria than any other kidney structure, more active transport mechanisms than other types of renal cells are required to reabsorb 80% of the filtrate that passes through the glomerulus, including glucose, ions, and the nutrients. The proximal tubule, the loop of Henle, the distal tubule, and the collecting duct require active transport to reabsorb the ions. Proximal tubular epithelial cells are rich in mitochondria and depend on oxidative phosphorylation for the generation of ATP [63]. The ability of mitochondria to detect and respond to energy demand and changes in nutrient availability through the maintenance of mitochondrial homeostasis is essential for the proper functioning of the proximal renal tubules [64]. In the late 1990s, Xie and Askari published studies that indicated that the enzyme Na^+^/K^+^-ATPase not only acts as an ion pump but also interacts as a signal transducer with several signaling pathways. Since then, numerous studies have shown that Na^+^/K^+^-ATPase participates in the mediation of long-term physiological adaptation processes that Neal Bricker originally proposed in the 1970s. It has been shown that Na^+^/K ligand^+^-ATPase, like cardiotonic steroids, emits signals through Na^+^/K^+^-ATPase and is involved in mediating adaptive and nonadaptive responses to volume overload, fibrosis, and oxidative stress [65]. Long-term hyperuricemia has the ability to induce hypertension, renal vasoconstriction, tubular damage, oxidative stress of the renal cortex, mitochondrial dysfunction, and decreased ATP levels [66].

### 1.5. Mitochondrial Dysfunction in CKD

The so-called “unifying hypothesis” is based mainly on the notion that mitochondrial dysfunction mediated by ROS overproduction is a common pathway in the pathogenesis of microvascular complications of diabetes mellitus that include diabetic nephropathy and progression to CKD [67]. The hypothesis does not explain all related phenomena, including ischemia as a common factor in CKD capable of producing mitochondrial inflammation and loss of membrane ridges in proximal podocytes and tubules [68]. The persistent accumulation of damaged mitochondria conditions deterioration of mitochondrial quality control [69]. Many mitochondria show evidence of proteolytic degradation that affects the mitophagy and favors the elimination of proteins damaged by mitochondrial proteases [70]. In experimental studies of CKD, fatty acid oxidation defects have been reported. Apparently, the high-fat diet causes the accumulation of lipids in the tubular cells and downregulates cathepsin-1 [71]. Defective fatty acid oxidation is frequently observed in patients with diabetic nephropathy. In a previous report, an increase in lipid deposition was reported and intracellular lipid drops in renal biopsies [72]. Lipid deposition is associated with negative regulation of several key genes involved in the oxidation of fatty acids. Transcriptome analysis of the entire genome of a large cohort of fibrotic kidney biopsies revealed low expression of cathepsin-1, cathepsin-2, and other genes involved in the metabolic pathways of fatty acid and glucose oxidation [73]. *In vitro* inhibition of fatty acid oxidation in tubular epithelial cells decreases the synthesis of ATP and produces metabolic alterations similar to those observed in CKD [73].

### 1.6. Oxidative Stress and Cardiolipin

Peroxidation of phospholipid fatty acids is presented as the main cause of oxidative signaling; therefore, deregulation of these pathways can lead to the accumulation of excessive amounts of lipid oxidation products at specific sites [74]. Several enzymes of the Krebs cycle, such as 2-oxoglutarate dehydrogenase (OGDH), branched chain complex-2-oxoacid dehydrogenase (BCKDH), and pyruvate dehydrogenase (PDH), are capable of producing considerable production of O_2_^·-^ and H_2_O_2_. BCKDH can produce O_2_^·-^ and H_2_O_2_ at higher rates compared to mitochondrial complexes I and III, which represents an additional source of ROS [75, 76]. Enzymatically catalyzed oxidation reactions can affect specific phospholipid molecules capable of leading to activation of the intrinsic mitochondrial apoptotic pathway. It has been reported that phospholipid-specific oxidation is one of the characteristic features of oxidation reactions triggered by different stimuli in different tissues [77]. The oxidation process of cardiolipin is not random; it has pronounced specificity not related to the polyunsaturation pattern of cardiolipin. The accumulation of hydroxyl derivatives and cardiolipin hydroperoxide in the tissues of affected animals has been observed. The above suggests that there are specific enzymatic mechanisms, such as the peroxidase activity of the cyt c/cardiolipin complex, which could be primarily responsible for the proapoptotic production of death signals [78]. The peroxidation of cardiolipin causes its translocation to the external mitochondrial membrane, where it has been proposed that it serves as a coupling site for the assembly of the inflammasome, while mitochondrial ROS activate the inflammasome to produce caspase-1 [79].

### 1.7. Cardiolipin, Inflammation, and Innate Immunity

The innate immune system is implicated in acute and chronic kidney injury [80]. Necrotic cells release damage-molecular patterns (DAMP) which can trigger an inflammatory form of cell death mediated by Nod-like receptors (NLRs) [81]. Several NLRs form a multimeric protein complex called the inflammasome, of which NLRP3 is best understood for its involvement in acute and CKD [82]. The NLRP3 inflammasome activates caspase-1 that causes the secretion of IL-1*β* and IL-18 proinflammatory cytokines and is considered a final common pathway that supports the cycle of inflammation and fibrosis in CKD. IL-1*β* and IL-18 represent possible therapeutic targets for CKD [83]. The inflammasome is activated in response to various stimuli that seem to converge on mitochondrial ROS generation [61]. NLRP3 binds directly to cardiolipin, suggesting that mitochondrial damage and translocation of cardiolipin to the outer mitochondrial membrane may provide the coupling signal for the assembly and activation of the inflammasome [84]. The continued presence of externalized cardiolipin in mitochondria damaged by the failure of its effective elimination by the innate immune system or by constant production because of cell death activates antigen-presenting cells (dendritic cells and macrophages) through interactions with T and B cells. They have the ability to stimulate the adaptive immune response that results in the production of ACA [85]. The effects of cardiolipin can be increased with beta-2-glycoprotein 1 (*β*2GPI). *β*2GPI is a glycoprotein present in high concentrations in plasma with the ability to bind effectively to anionic phospholipids (including cardiolipins). Excessive formation and accumulation of APA, particularly ACA, may condition the development of a systemic autoimmune disease [86]. Cardiolipin complexes with cyt c that produce proapoptotic signals catalyze the oxidation of the polyunsaturated acyl chains of cardiolipin. Countless cardiolipin species exert messaging functions and oxidation products with important intra- and extracellular signals for innate and adaptive immunity systems [87]. Correlation between mitochondrial DNA (mDNA) levels in donor plasma and early allograft dysfunction in liver transplant recipients has been demonstrated, suggesting the potential role of circulating mtDAMP in allograft results. The current advisable approach to prolonging allograft survival in addition to immune suppression in the transplant recipient may be to attack inflammatory factors in donors (deceased) before obtaining as a potential strategy to improve transplant results [88]. Several primary and systemic kidney diseases are associated with the activation of the NLRP3 inflammasome/IL-1*β*/IL-18 axis. The spectrum of the disease includes ureteral obstruction, ischemia/reperfusion injury, glomerulonephritis, sepsis, hypoxia, glycerol-induced renal failure, and crystalline nephropathy. In addition, the IL-1/IL-18 axis may be responsible for the development of CKD and its related complications [83].

### 1.8. Mitophagy and Cardiolipin

The collapse of asymmetry in the distribution of lipids in the mitochondrial inner membrane represents a promitophagy mechanism by which externalized cardiolipin acts as a signal to initiate mitophagy [46]. The autophagic machinery releases damaged mitochondria in double-membrane vesicles (autophagosomes), which fuse with late endosomes or lysosomes so that lysosomal enzymes degrade the autophagosome content, including the internal autophagosome membrane [89]. Receptors or selective autophagic loading adapters (p62 and optineurin) unify their mitochondrial load attached to the autophagosome membrane by interacting with the lipid protein (phosphatidylethanolamine ATG8, microtubule-1-associated protein (LC3))/protein associated with the amino acid gamma butyric receptor (GABARAP) intimately associated with autophagosome membrane charge receptors [90]. Autophagy LC3 protein mediates autophagosome formation and load recognition because it contains important cardiolipin binding sites to facilitate thickening of mitochondria [91]. In normally functioning mitochondria, cardiolipin is limited to the internal mitochondrial membrane (predominantly oriented to the matrix near the site of its synthesis). Recognition by cytosolic LC3 requires the presence of cardiolipin on the mitochondrial surface, suggesting that transmembrane redistribution of cardiolipin should be performed in damaged mitochondria that undergo mitophagy. In mitochondria that maintain their electrochemical membrane potential, cardiolipin binds directly to many membrane proteins and respiratory complexes and supercomplexes. Negligible amounts of cardiolipin bind to soluble proteins [92]. Previously, the general dependence of mitophagy and the externalization of cardiolipin on the total cardiolipin content have been established. The mechanisms of translocation of cardiolipins within mitochondrial membranes have not yet been completely deciphered. It is suspected that decoupling proteins, anionic transporters of the internal mitochondrial membrane and phospholipid scramblase-3, may participate in this phenomenon [30]. Association of autophagy with the pathogenesis of various diseases has been reported: neurodegeneration, cancer, infections, and kidney diseases. Proximal tubules potentially require a lot of energy to degrade. Apparently, autophagy plays an important role in the physiology of the proximal tubules. Autophagy has been reported to have a protective role against damage to the proximal contoured tubules by the ischemia/reperfusion and nephrotoxic processes, including cisplatin and cyclosporine [93].

### 1.9. Anticardiolipin Antibodies

High prevalence of ACA (19%) is reported in CKD patient candidates for RT compared to the general population due to the association of ACA with autoimmune diseases. Patients with CKD are more likely to suffer from comorbid conditions (infections, aging, and the use of nephrotoxic medications) [94]. Patients without a history of coagulation disorders have no transplant thrombosis compared to those with APA-positive diagnosis [95]. ACAs can also be detected after bacterial or viral infections. Among patients receiving dialysis, having a positive antibody concentration was not associated with age or having received RT. The presence of ACA was more common among patients with chronic glomerulonephritis than among those with other diagnoses [89].

The incidence of acute rejection leading to graft failure in patients with ACA but not APA is considerably high (27%) in contrast to the group of patients without ACA and with APA (14%) (*p* < 0.05) vs. healthy controls [96]. ACA-positive patients may have a better immune response. Patients with APA syndrome who do not receive anticoagulation before RT have an incidence of 100% of graft thrombosis with graft insufficiency [97]. Anticoagulation before or at the time of RT in patients with APA significantly reduces the incidence of posttransplant thrombosis and graft failure [70]. None of the ACA isotypes is specific for the development of thrombosis in patients with APA syndrome and ESRD. Several authors have noted that APA thrombosis is associated with ACA of the IgG, IgM, and IgA isotypes [98]. The nature of the target molecules for ACA is unclear. It has been reported that there may be more than one candidate molecule (*β*2 glycoprotein and/or phosphatidylserine) [73]. The mechanisms underlying vascular thrombosis remains uncertain; however, it has been postulated that the surgical procedure and endothelial cell damage can act synergistically in patients with APA to induce the thrombotic process in allografts [99]. Several authors have shown that alloantibodies against endothelial and/or epidermal antigens can cause acute accelerated loss of the graft [100] (Table 1).

### 1.10. Antiphospholipid Antibodies

APAs are a heterogeneous group of autoantibodies present in a wide range of diseases. Among the APAs, those directed against cardiolipin and its cofactor (*β*2-GP1) are best characterized and used in clinical practice. APA produces the antiphospholipid syndrome [101]. APAs are autoantibodies associated with venous and arterial thrombosis [102]. The presence of antithrombus and/or anticardiolipin antibodies indicates a worse long-term renal prognosis. ACA positivity is a strong predictor of venous and arterial systemic vascular thrombotic complications [103]. APAs are a heterogeneous group of immunoglobulins directed against negatively charged phospholipids, protein-phospholipid complexes, or plasma *β*2-GPI [104]. APAs include ACAs and lupus anticoagulant (LAC). ACAs are recognized for their high ability to bind to anionic phospholipids in solid-phase immunoassays with the ability to lengthen phospholipid-dependent coagulation tests [19]. APAs can interfere with the atherogenic process and crossreact with low-density lipoprotein (LDL) antioxidant antibodies (Table 1).

### 1.11. Cardiolipin Inhibition of Platelet-Activating Factor Could Lead to Thrombosis

Platelet-activating factor (PAF) (1-0-alkyl-2-acetyl-sn-glycero-3-phosphocholine) is a natural lipid mediator with a wide range of *in vivo* and *in vitro* effects including pathological responses in inflammation and allergy. PAF has the ability to stimulate platelets, neutrophils, macrophages, and endothelial cells that induces platelet aggregation and activation of the coagulation system [105]. Cardiolipin is a specific inhibitor of PAF [106]. It is possible that the vascular thrombosis observed in HD patients, as well as in ACA or APA syndrome, is the result of the inactivation of circulating ACA that leads to increased PAF activity. Thrombosis in patients undergoing HD could be the result of the action of PAF in platelets and the activation of the coagulation cascade [107]. This topic is interesting as a diagnostic target.

### 1.12. Cardiolipin and Nephropathy

Cardiolipin is essential for the formation of the crystalline membrane and organizes the components of the electron transport chain, preserving the integrity and mitochondrial structure. The metabolic syndrome increases more saturated species such as C18:1, C18:0, and hydrolysis products of renal cardiolipin (lysocardiolipin), which implies the oxidation of cardiolipin [108]. Renal adiposity, lipid peroxidation, and elevated levels of isoprostanes seen in mitochondrial syndrome probably contribute to renal mitochondrial injury by mitochondrial cardiolipin peroxidation [109]. Patients with uremia have a high incidence of infectious, cardiovascular, and thrombotic events caused by ACA. In dialyzed patients, ACA levels are higher than those in healthy subjects without association who have received a previous RT. It is more common to find ACA in patients with glomerulonephritis than in patients undergoing RT [110]. ACAs are clinically associated with hypercoagulability. Vascular access thrombosis and systemic thrombosis in HD coexist with immunoregulation abnormalities in ESRD [86]. IgG-ACA is strongly associated with the presence of venous and arterial thrombosis in patients with normal renal function. High titers of IgG-ACA have also been reported in HD patients [111].

### 1.13. Cardiolipin and Kidney Transplant

It is well known that CVD is more common among RT recipients than in the general population and is the leading cause of death [112, 113]. One of the characteristics of APA syndrome is the presence of ACA in association with thrombotic disorders or thrombocytopenia of the arterial and/or venous systems [114]. Since the 1990s, increased serum levels of ACA have been reported in HD patients and in RT recipients, with a prevalence of 4.8 to 46.4%. The clinical importance of ACA positivity in these patients was uncertain. Serum IgG and IgM-ACA levels of 61 patients were measured in HD. 14 were RT recipients and 38 were healthy controls. The authors reported increased levels of ACA-IgG in four HD patients, in two RT (14%) and two healthy controls. After one year of follow-up, serum ACA-IgM levels were normal in all patients without observing events [115]. Acquired thrombophilia is specifically considered for the presence of APA with the ability to predispose to adverse outcomes after transplantation, although this association is not clear [96]. ACAs are by far the most common prevalence in ESRD which are considerably higher than those in healthy controls. Primary and secondary APA positivity is associated with specific intrarenal changes characterized by fibrous intimal hyperplasia, focal cortical atrophy, and thrombotic microangiopathy [116]. Several published series have reported ACA in 10-30% of dialysis patients in contrast to 1-8% of the general population [117]. Similar findings have been reported of the presence of APA in patients with RT [118].

Inflammatory phenomena secondary to episodes of acute RT rejection may be responsible for *de novo* production of APA by contributing to the development and progression of CVD [119]. In this context, cell death occurs due to necrosis or apoptosis for the exposure of the immune system to autoantigens towards which there is no tolerance [119]. Patients with ESRD with APA are at high risk of developing renal thrombosis within a period as short as 1 week after transplantation. When the thrombosis process begins, no treatment protocol that destroys the clot to save the RT is available [120]. Therefore, prevention of thrombosis is of the utmost importance; it is required that potential transplant recipients be diagnosed with APA or ACA before transplantation so that anticoagulant therapy can be initiated before transplantation. Thrombosis that occurs in renal grafts shortly after kidney transplantation in the absence of prior systemic coagulopathy is considered a result of acute rejection or immunologically induced coagulopathy [121]. Previous fibrin deposition has been reported in failed grafts without evidence of systemic coagulopathy, indicating that thrombosis is limited to the graft [122]. Thrombosis can occur up to the second week after transplantation and represents 12.2% of failed primary transplants and 19.2% of failed repeat transplants [123]. The effects of APA include interference with the atherogenic process by crossreacting with antibodies against oxidized low-density lipoprotein, improved uptake of oxidized LDL macrophages, and promotion of thrombosis [124]. It is well known that stable RT recipients have disproportionately high rates of atherosclerosis, where APA may apparently be involved [125]. Moreover, the presence of APA is considered an independent cardiovascular risk factor in RT recipients [113]. The evaluation of APA at the time the patient appears as a candidate for RT facilitates the identification of patients at high risk of early graft dysfunction. In addition, predisposition to thrombotic complications after RT has been identified in patients with a history of deep venous thrombosis before transplantation. RT candidates with a history of one or more thrombotic events may have increased risk of APA systemic vascular thrombosis [126]. The pretransplant determination of each patient's APA *status* can predict which patients may benefit from prophylactic anticoagulant therapy to prevent graft thrombosis [127]. Previous reports indicate that most APA may be acquired in the pretransplant period [128]. More than half of patients with APA before transplantation lost these antibodies after transplantation [129]. However, the persistence of APA identified before transplantation in the posttransplant period is associated with excessive risk of atherosclerotic events [113]. In fact, patients who never had APA and those who lost APA before transplantation during the posttransplant period seem to have a lower risk of atherosclerotic events. This result is important for the search of APA and the management of patients in the transplant period [116]. Prophylactic measures are not likely to be indicated in all APA-positive patients during the initial period after RT. These measures should be analyzed if the APA positivity persists during the months after the transplant [130]. APA syndrome is one of the differential diagnoses for thrombotic microangiopathy that can cause acute loss of graft function or fatal thrombotic complications. A patient with polycythemia was previously reported after RT incompatible ABO and negative APA screening tests before transplantation. The patient was treated with desensitization therapy (plasmapheresis and rituximab); however, he developed acute graft dysfunction on day 112 postoperatively. Graft biopsy revealed microvascular inflammation of the glomerular capillaries without immunoglobulin deposition. The ACA was positive repeatedly without previous thrombotic episodes. The treatment was based on intensive steroid pulse, plasmapheresis, and induction with rituximab. The ACA disappeared, and the graft function recovered and stabilized for 52 months. The authors suggest that asymptomatic ACA may be associated with acute graft dysfunction [9].

### 1.14. Cardiolipin in RT-Related Diseases

Bone mineral disorders frequently occur in RT recipients and are associated with a high risk of fractures, morbidity, and mortality. The pathophysiology underlying bone disorders after RT results from a complex interaction of factors, including preexisting renal osteodystrophy and bone loss related to immunosuppression and alterations in the growth factor of fibroblast 23-parathyroid hormone-vitamin D [131]. The neurological complications observed in solid organ transplants are reported in almost 9 of 10 recipients. The intensity, severity, and type of anomalies may vary. More often, complications appear to be associated with the direct or indirect effect of immunosuppressants because of their direct effect on cells and blood vessels and susceptibility to infections [132]. However, we found no information in the available literature on the complications of RT related to cardiolipin and its antibodies. We believe that this could be an interesting topic to investigate in RT recipients.

### 1.15. Elamipretide and Cardiolipin

Antioxidants are considered protective molecules against oxidative stress by acting predominantly through mechanisms of elimination of free radicals [133]. Alternative ideas related to enzymatically regulated oxidative reactions have been presented, including phospholipid peroxidation reactions as the main cause of oxidative signaling. Deregulation of these reactions can lead to the accumulation of excessive amounts of lipid oxidation products capable of exacerbating oxidative damage in specific molecular targets [56]. Apparently, phospholipids are oxidized in a manner dependent on the amount of double bonds in their acyl groups, rather than the nature of their polar group [134]. Elamipretide (SS-31) is a cationic synthetic tetrapeptide (D-Arg-2′6′-dimethyl Tyr-Lys-Phe-NH2) that specifically binds with high affinity to cardiolipin in the inner mitochondrial membrane. SS-31 favors electron transfer of cyt c to cyt c oxidase and minimizes ROS production. Another proposed mechanism of the SS-31 is based on the protection of the highly curved structure of the mitochondrial ridges facilitating the transport of electrons and minimizing the production of ROS [109]. This tetrapeptide has antioxidant properties and inhibits the peroxidase activity of the cyt c-cardiolipin complex, which inhibits the opening of the mPTP [109]. In a recent experimental study, it was reported that treatment with elamipretide restored renal function in swine reno-vascular disease [135]. A recent study showed that elamipretide prevents glomerulopathy induced by a high-fat diet and a proximal tubular lesion in mice [136]. In 2017, a study was reported in which SS-31 was administered in experimental animals. Administration began 1 month after producing ischemia and was maintained for 6 weeks. The authors observed protection of mitochondrial integrity, and the progression of glomerulosclerosis and tubular interstitial fibrosis was avoided. Protection by SS-31 was maintained for 6 months after the end of treatment [136]. In addition, there was positive regulation of IL-1*β* and IL-18 nine months after ischemia, suggesting that SS-31 reduced CKD by protecting mitochondria and preventing the activation of the inflammasome [51]. Cardiolipin promotes mitochondrial ridge formation, promotes normal assembly of respiratory complexes to form supercomplexes, facilitates efficient electron transfer, promotes ATP synthesis, and reduces electron leakage [136]. Dysfunctional mitochondria generate ROS, and cardiolipin is particularly susceptible to lipid peroxidation. In the presence of ROS, cyt c acts as peroxidase and produces peroxidation of cardiolipin, which causes degradation of mitochondrial ridges [43]. Previously, it has been reported in experimental models of renal and cardiac insufficiency that SS-31 restores cardiolipin content and mitochondrial ridge structure and improves mitochondrial energy [137]. The release of mitochondrial DNA, ROS, and cardiolipin from damaged mitochondria can activate the NLRP3 inflammasome. The administration of SS-31 promotes bioenergetic recovery and the structure of the podocytes allowing the repair of the actin cytoskeleton [138]. In addition, SS-31 prevents positive regulation of IL-1*β* and IL-18 after acute renal ischemia as previously mentioned [139].

In conclusion, cardiolipin is a fundamental component of the total phospholipids of body tissues. Cardiolipin is characteristically associated with the internal mitochondrial membrane, where it plays an important biological role in favoring the high rate of ATP synthesis. The ionization state of cardiolipin influences the interaction with other molecules of the internal mitochondrial membrane. In addition, *in vitro* studies suggest that cardiolipin induces the formation of dynamic narrow tubular extensions or invaginations similar to mitochondrial ridges capable of changing shape depending on the pH of the medium. Therefore, by keeping cardiolipin the highly curved structure of mitochondrial ridges, it modulates essential mitochondrial enzymatic activities such as those of F_0_F_1_-ATP synthase, ATP/ADP transporter, and decoupling protein and electron chain transport, among others. APA and ACA are heterogeneous groups of autoantibodies involved in various pathologies. In patients with CKD, candidates for RT, there is a high prevalence of ACA (19%) and the incidence of acute rejection of RT in patients positive for ACA but negative for APA is 27%. The above suggests that ACA-positive patients have an intensified immune response, which could condition renal graft loss. Patients with ESRD and ACA or APA positivity have a high risk of developing venous or artery renal thrombosis shortly after RT. The management of the thrombosis process in these patients is very difficult and jeopardizes the viability of the transplanted graft. It is convenient to determine the presence of ACA or APA in RT candidates; in the positive case, the appropriate antithrombotic treatment was provided to prevent graft loss. The determination of cardiolipin or its antibodies is an attractive therapeutic, diagnostic target in RT and kidney diseases.

## Figures and Tables

**Table 1:** Features of ACA and APA antibodies in the kidney.

ACA	APA
Antibodies associated with hypercoagulability In hemodialysis, systemic thrombosis and vascular access thrombosis coexist [86] High incidence of infectious, cardiovascular, and thrombotic events Antibodies more frequent in those with glomerulonephritis than in those undergoing kidney transplantation Thrombotic disorders/thrombocytopenia of the arterial and/or venous systems [86] Disproportionately high rates of arteriosclerosis [91] High risk of developing renal thrombosis in 1 week after transplant, thrombosis can be limited to the graft [46] Thrombosis can occur in the second week after transplantation and represents 12.2% of failed primary transplants and 19.2% of failed repeated transplants [89]	Venous thrombophilic and arterial autoimmune condition [1] Primary; absence of another related disease. The initial presentation may be with vasculitis [2]. The kidney can be the initial target [3] Secondary to another concurrent autoimmune disease The presentation may vary; it mimics many other medical conditions Kidney clinical manifestations [4] Hypertension, microscopic hematuria, proteinuria (from mild to nephritic levels), renal insufficiency, thrombosis or stenosis of the renal artery, thrombosis of the renal vein, graft loss due to thrombosis after kidney transplantation, injury of the renal microvasculature (renal nephropathy) APA Kidney biopsy [5] Acute lesions of thrombotic microangiopathy, chronic intrarenal vascular lesions, intimal fibrous interlobular hyperplasia, recanalizing arterial and arteriolar thrombosis, fibrous arterial occlusion, and focal cortical atrophy
